# Transient elevation of glycolysis confers radio-resistance by facilitating DNA repair in cells

**DOI:** 10.1186/s12885-015-1368-9

**Published:** 2015-05-01

**Authors:** Anant Narayan Bhatt, Ankit Chauhan, Suchit Khanna, Yogesh Rai, Saurabh Singh, Ravi Soni, Namita Kalra, Bilikere S Dwarakanath

**Affiliations:** Metabolic and Cell Signaling Group, Institute of Nuclear Medicine and Allied Sciences, Brig SK Mazumdar Road, New Delhi, Delhi 110 054 India

**Keywords:** DNP, Respiratory modifiers, Glycolysis and radio-resistance

## Abstract

**Background:**

Cancer cells exhibit increased glycolysis for ATP production (the Warburg effect) and macromolecular biosynthesis; it is also linked with therapeutic resistance that is generally associated with compromised respiratory metabolism. Molecular mechanisms underlying radio-resistance linked to elevated glycolysis remain incompletely understood.

**Methods:**

We stimulated glycolysis using mitochondrial respiratory modifiers (MRMs viz. di-nitro phenol, DNP; Photosan-3, PS3; Methylene blue, MB) in established human cell lines (HEK293, BMG-1 and OCT-1). Glucose utilization and lactate production, levels of glucose transporters and glycolytic enzymes were investigated as indices of glycolysis. Clonogenic survival, DNA repair and cytogenetic damage were studied as parameters of radiation response.

**Results:**

MRMs induced the glycolysis by enhancing the levels of two important regulators of glucose metabolism GLUT-1 and HK-II and resulted in 2 fold increase in glucose consumption and lactate production. This increase in glycolysis resulted in resistance against radiation-induced cell death (clonogenic survival) in different cell lines at an absorbed dose of 5 Gy. Inhibition of glucose uptake and glycolysis (using fasentin, 2-deoxy-D-glucose and 3-bromopyruvate) in DNP treated cells failed to increase the clonogenic survival of irradiated cells, suggesting that radio-resistance linked to inhibition of mitochondrial respiration is glycolysis dependent. Elevated glycolysis also facilitated rejoining of radiation-induced DNA strand breaks by activating both non-homologous end joining (NHEJ) and homologous recombination (HR) pathways of DNA double strand break repair leading to a reduction in radiation-induced cytogenetic damage (micronuclei formation) in these cells.

**Conclusions:**

These findings suggest that enhanced glycolysis generally observed in cancer cells may be responsible for the radio-resistance, partly by enhancing the repair of DNA damage.

## Background

Ionizing radiation plays an important role in the management of a majority of malignancies [1], although many tumors like glioma and several carcinomas are known to be refractory to radiotherapy with marginal benefits in survival. However, the molecular mechanisms underlying this radio-resistance of cancer cells remain poorly understood. One of the most common signatures of highly malignant tumors is their capacity to metabolize more glucose to lactic acid than normal tissues, which confers a selective growth advantage [2]. Cells derived from hypoxic tumors typically maintain their metabolic phenotypes even under normoxic culture conditions (Warburg effect), indicating that aerobic glycolysis is constitutively upregulated through stable genetic or epigenetic changes [2]. It is also reported that mitochondrial defect linked stabilization of HIF1α induces glycolytic phenotype in cancer cells and promotes aggressiveness of tumors [2,3]. On the other hand, efficient oxidative phosphorylation in cancer cells is required for execution of apoptosis through the generation of reactive oxygen species (ROS) [4]. Therefore, metabolically reprogrammed and highly glycolytic cancer cells can easily escape the death processes, conferring resistance to therapeutic modalities [5].

The phenotypic characteristics of enhanced glycolysis associated with tumors have been well exploited for the diagnosis of the disease using fluoro-deoxy glucose (FDG) based positron emission tomography (PET) imaging and the efficacy of glycolytic inhibitors as sensitizers to radiation and chemotherapeutic drugs has established in pre-clinical studies, while clinical trials are at different stages of evaluation [6-9]. Considerable amount of evidences suggest that inhibition of glycolysis leads to compromised DNA repair, which is accompanied by the depletion of energy [ATP and AXP (AMP and ADP)] in cells with high rates of glycolysis like the cancer cells, causing death [10-14]. However, the mechanisms underlying enhanced resistance to radiation-induced cell death in cells with high endogenous rates of glycolysis, like the cancer cells are not completely understood, although alterations in pH and lactate levels have been implicated [2]. Therefore, present studies were undertaken to examine if transient stimulation of glycolysis (before irradiation) using MRMs is sufficient to confer radio-resistance and also to unravel the underlying mechanisms. Results obtained in human malignant and non-malignant cell lines clearly show that stimulation of glycolysis using MRMs reduces radiation-induced cell death by enhancing the repair of DNA damage leading to a reduction in the mitotic death linked to cytogenetic damage (micronuclei formation).

## Methods

### Materials

Dulbecco’s Minimum Essential Medium (DMEM), Penicillin G, Streptomycin, Nystatin, Dimethyl sulfoxide (DMSO), Dichlorodihydrofluorescein diacetate (H_2_DCFH-DA), Potassium chloride (KCl), Magnesium chloride (MgCl_2_), Ethylene-di-amine-tetra acetate (EDTA), Protease inhibitor cocktail, HEPES, Hoechst-33258 (H258), propidium iodide (PI), RNAase, Di-nitrophenol and methylene blue were procured from Sigma chemicals Co. (St Louis, USA). Photosan (PS3) was procured from See Lab, Germany. Bicinchoninic acid (BCA, Thermo fisher scientific Rockford USA), primary and HRP conjugated secondary antibodies were procured from Santa Cruz, USA.

### Methods

#### Cell culture

The cerebral glioma cell line (BMG-1; diploid, wild type p53) established by us earlier [12] whereas; OCT-1 (oral carcinoma) and HEK293 (human embryonic kidney) cells were obtained from NCCS, Pune, India. BMG-1 and OCT-1 cells were maintained as monolayers in Dulbecco’s modified Eagles medium (DMEM) supplemented with fetal bovine serum (5% for BMG-1 and 10% for OCT-1), HEPES and antibiotics as described earlier [12]. HEK293 was grown in high glucose (4.5 g/L) DMEM supplemented with 10% fetal bovine serum (FBS). All experiments were carried out in exponentially growing cells.

#### Treatments

Cells were treated with MRMs at concentrations that induce glycolysis without any cytotoxicity (data not shown). The drug concentrations used in all the experiments were 1 μM for DNP, 25 μg/ml for PS3 and 25 μM for MB. Cells were exposed to 5 Gy γ-ray irradiation in all experiments (unless specified otherwise in particular experiments), using Bhabhatron-II, a teletherapy machine from Panacea, Medical Technologies Pvt. Ltd. (Bangalore, India) at 80 cm SSD and 35x35 cm field with a dose rate of 2 Gy/min.

#### Glucose consumption and lactate production assay

BMG-1, OCT-1 and HEK293 cells were incubated in HBSS or HBSS containing MRMs before irradiation. Cells were incubated for 4 hours; every hour HBSS sample was taken from dishes and frozen immediately for further analysis. The amount of glucose remaining unused and the lactate produced were estimated in the HBSS using enzymatic assays. Glucose was determined by the glucose-oxidase method using Tecnicon RA-500 auto analyzer. Lactate was estimated using lactate oxidase method based kit (Randox; Cat. No.-LC2389). Glucose consumption and lactate production were estimated at the end of every hour (up to four hours) and presented as the average consumption or production per hour. The number of viable cells was counted on hemocytometer using trypan blue (0.4%) exclusion test and glucose consumption or lactate production was normalized with respect to the number of trypan blue negative (viable) cells.

#### Hexokinase assay

Hexokinase enzymatic activity was measured as previously described [15]. Briefly, cells were treated for 4 hour with DNP followed by radiation before washed with PBS and lysed using the buffer containing 50 mM Tris-HCl (pH-8.0), 1.5 mM MgCl_2,_ 150 mM NaCl, 250mM sucrose along with protease inhibitor. The cell lysate was incubated on ice for 30 min, followed by centrifugation at 14,000 rpm at 4°C for 10 min. The protein estimation was done by BCA method. An aliquot of 20 μg protein from freshly lysed cell supernatant was added to 1 ml of reaction buffer containing 100 mM Tris-HCl, pH 8.0, 0.5 mM EDTA, 10 mM ATP, 10 mM MgCl_2_, 2 mM glucose, 0.1 mM NADP, and 0.1 U/ml of G6PD (Sigma). HK activity was determined by following the G6P-dependent conversion of NADP to NADPH spectrophotometrically at 340 nm at room temperature. The values were subtracted with respective blanks and the relative enzymatic activity is presented as absorption at 340 nm.

#### ATP measurement

ATP was measured using ATP bioluminescent assay kit (Sigma) following manufacturer’s protocol. Briefly, cells were treated with MRMs followed by irradiation for 4 hour before washed and scraped in cold PBS and pelleted at 1000 rpm for 10 min. Further, cells were lysed in 350 μl of lysis buffer (4mM EDTA and 0.2% Triton X-100). 100 μl of this lysate was loaded per well in triplicates with 100 μl of ATP mix in a 96-well white luminescence measuring plate. Luminescence of samples along with standards was read at 562 nm and normalized with the protein concentration. ATP concentration is depicted as ng/mg protein.

#### Measurement of mitochondrial content and membrane potential

Cells were treated with MRMs followed with or without irradiation for various time points. Once the incubation time was completed, cells were washed with PBS and stained for 30 minutes at 37°C in CO_2_ incubator with either 100 nM mitotracker green or 100 nM DiOC6 (both from Molecular Probes, Invitrogen) in respective wells. After staining, cells were trypsinized and equal numbers of cells were transferred to 96 well fluorescence reading plate. The fluorescence was measured at 490 nm excitation and 516 nm emission for mitotracker green and 484 nm excitation and 501 nm emission for DiOC6 in quadruplicates using Molecular device fluorescence plate reader. The fluorescence intensity of mitotracker green is represented as relative mitochondrial content and the DiOC6 fluorescence intensity is represented as relative mitochondrial membrane potential.

#### Macro colony assay

Clonogenic survival was performed using post plating method of macro colony assay. Cells were plated at a density of 6,000 - 8,000 cells/cm^2^ in Petri dishes. 36 h later, cells were washed with HBSS and treated with different MRMs (viz. 1 μM di-nitro phenol, DNP; 25 μg/ml Photosan-3, PS3; 25 μM Methylene blue, MB) in HBSS before irradiation. Following the radiation treatment, cells were incubated for 4h at 37°C in HBSS in CO_2_ incubator then washed twice with HBSS, trypsinized, counted and plated in triplicates in 60 mm petri dish at very low density (100 cells/dish) for control and MRMs treatment alone and relatively high cell density (500 cells/dish) for radiation (5 Gy) and MRMs with radiation. These dishes were incubated in fresh media at 37°C in a humidified CO_2_ (5%) incubator for 7-10 days, depending on the cell line. Cultures were terminated when macrocolonies were visible after 7-10 days, were fixed with 10% methanol in PBS and stained with 1% crystal violet (dissolved in 7% methanol in PBS). Colonies of at least 50 cells (5 to 6 doubling) were scored as survivors. Plating efficiency was calculated as: PE = (No. of colonies counted/No of cells plated) x 100. The surviving fraction was calculated as: SF = PE_T_ /PE_C._ Where PE_T_ is the plating efficiency of the treated group and PE_C_ is the value of the control.

#### Single cell gel electrophoresis (comet assay)

Neutral comet assay was performed as described earlier [16,17]. For 0 hour time point (to obtain damage induction) cells were exposed on ice and processed immediately after exposure. Briefly, treated cells were trypsinized and resuspended in the medium at respective time points. An aliquot of 30,000-40,000 cells (~100 μl of medium) was mixed into a suspension of 0.75% (~500μl) warm low gelling (gelling temp = 17°C) agarose (BDH Electran, England), and spread onto microscopic slide (pre-coated with 0.1% agarose) kept at 45°C. Slides were immediately transferred to a cooling plate at 4°C, and kept for 5 min to allow the agarose layer to gel along with the embedded cells. Following this, the slides carrying agarose gel with embedded cells were immersed in the SDS lysis buffer [2.5% sodium dodecyl sulphate (SDS), 1% sodium sarcosinate and 25 mM ethylenediaminetetraacetic acid (EDTA)] for 15 min at room temperature (25-28°C). Slides were then washed in double distilled water for 5 min at 4°C, electrophoresed at 2 V/cm for 5 min, and air-dried at 45°C. For analysis, the slides were rehydrated by immersing in distilled water at room temperature, and stained with 25 μg/ml propidium iodide (PI) dye. Comet-like shape forms from the DNA mass of each individual cell following these treatments, the amount of DNA present in the tail directly corresponding with the amount of DNA breaks induced by radiation treatment. The comets were randomly selected (n = 50) and images were acquired at 200x under BX60 fluorescence microscope (Olympus, Japan). The comets were analyzed using OPTIMAS image analysis software (calibrated for analyzing comets) and the tail moment was calculated from data generated by the software.

#### Micronuclei assay

Cells were washed in PBS and fixed in carnoys fixative (3:1 V/V, Methanol: Acetic acid). Fixed cells were dropped on the chilled glass slides. Air-dried slides were stained with a DNA binding fluorochrome (bisbenzimidazole; hoechst-33258) at 10 μg/ml dissolved in citric acid [0.01 M], disodium phosphate [0.45 M] buffer containing 0.05% Tween-20 detergent; pH 7.4 as described earlier [6]. Slides were examined under fluorescence microscope using UV excitation filter. Cells containing micronuclei were counted from 1000 cells as described earlier [6,18].

#### Immuno-blot

Expression of key proteins involved in glucose catabolism and DNA repair viz. glucose transporters GLUT-1, GLUT-4, hexokinase-II (HK-II), PFK-1, HIF1α, Rad51, Ku-70 and loading control β-Actin was determined in control and treated cells by immunoblot analysis. Cells were harvested at various time points (showed in figure) and lysed in ice cold RIPA lysis buffer (Tris–HCl: 50mM, pH 7.4, NP-40: 1%, Na-deoxycholate: 0.25%, NaCl: 150 mM, EDTA: 1 mM, PMSF: 2 mM, protease inhibitor cocktail, Na3VO4: 1 mM, NaF: 1 mM). The protein concentration in the lysates was measured using BCA protein assay. Protein (40–50 μg) was resolved on 10–15% SDS–PAGE (depending on the molecular weight) and electroblotted onto PVDF membrane (MDI). The membrane was then incubated in 4% skimmed milk for 1 h followed by primary antibody incubation GLUT-1 (1:300), GLUT-4 (1:300), HK-II (1:200), PFK-2 (1:200), Rad51 (1:200), Ku-70 (1:150) from Santa Cruz Biotechnology, HIF1α (1:1000) from Cell Signaling Tech. and β-Actin (1:5000) from BD Biosciences for overnight. Membrane was washed and incubated with the appropriate HRP conjugated secondary antibody for 1h. After washing, the blots were developed using ECL chemiluminescence detection reagent (Biological industries, Israel). The signal was detected by ECL, and band intensities for each individual protein were quantified by densitometry, corrected for background staining, and normalized to the signal for β-Actin.

#### Statistical analyses

All the experiments were performed in triplicates or quadruplicates. Means and standard errors were computed. Student’s *t* test was performed to determine whether a significant difference exists between the groups.

## Results

### Mitochondrial respiratory modifiers induces glycolysis

**Figure 1:**
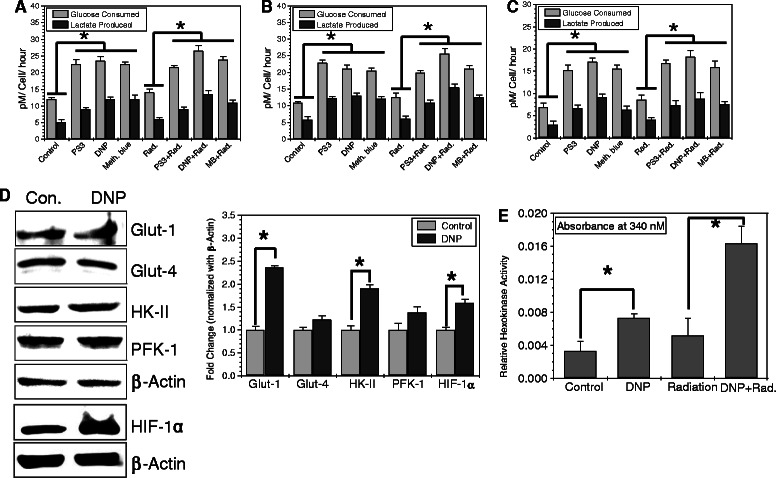
Mitochondrial respiratory modifiers (MRMs; PS3, DNP & MB) induces glycolysis. Glucose consumption and lactate production observed every hour till 4 hours of the drug treatment is presented as average per hour in BMG-1 **(A)**, OCT-1 **(B)** and HEK293 **(C)** cells. **(D)** Protein expression profile of glucose transporter, glycolytic enzymes and transcriptional regulator of glycolysis HIF1α is shown in BMG-1 cells. The data shows western blots and their derived quantitative values from the densitometry. **(E)** Relative hexokinase enzymatic activity in un-irradiated and irradiated (5 Gy γ-rays) BMG-1 cells is presented as absorbance at 340 nm obtained from coupled enzymatic assay. The concentration of different treatments used was as follows, PS3, 25 μg/ml; DNP, 1 μM; MB, 25 μM. The data shown are the mean values (±1 SD) of nine observations from three independent experiments. Statistical significance *p < 0.05.

To mimic the high glycolytic phenotype of cancer cells, we investigated the glycolysis stimulating potential of few mitochondrial respiratory modifiers (MRMs) that are known to stimulate glycolysis as a compensatory mechanism [19]. At Treatment of exponentially growing cells with non-toxic concentrations MRMs such as di-nitrophenol (DNP), porphyrin derivatives (photosan; PS3) and methylene blue (MB), which interfere with the oxidative phosphorylation at different stages in the electron transport chain (ETC), was found to enhance the glycolysis (glucose utilization and lactate production) significantly (by approximately two folds) in both malignant cell lines BMG-1 and OCT-1 (Figure 1A and B), similar to our earlier results with KCN [11,12]. To test if compromised oxidative phosphorylation can induce the compensatory increase in glycolysis in non-malignant cell similar to malignant cells, we treated HEK cell line (embryonic kidney) with MRMs under similar experimental conditions. Interestingly, MRMs induced the glucose uptake and lactate production in HEK cells also (Figure 1C). Further, we observed that irradiation alone also marginally increased glycolysis (Figure 1A, B and C) as reported earlier [11], with further increase in presence of MRMs (Figure 1A, B and C). It is pertinent to note that compensatory increase in glycolysis due to inhibition of oxidative phosphorylation appears to be not limited only to malignant cells.

To unravel the contributing factors responsible for MRM-induced enhancement in glycolysis, we examined the level of glycolytic enzymes and glucose transporters under similar experimental conditions. Interestingly, we found approximately 2.5 fold increased level of GLUT-1, while no significant change could be seen in GLUT-4 (Figure 1D). A 2 fold increase was also seen in the level of hexokinase-II, one of the first two regulatory kinases (HK-II and PFK-1) of glycolysis; however the level of PFK-1 does not change appreciably (Figure 1D). DNP treatment also showed increased level of hypoxia inducible transcription factor, HIF1α which is known to induce glycolysis. Further, the increase in hexokinase expression also correlated with nearly two fold increase in the total hexokinase activity (Figure 1E) induced by DNP under these experimental conditions. Interestingly, the hexokinase activity was increased further by nearly 4 fold in cells treated with both DNP and radiation. These findings suggest that inhibition of mitochondrial respiration stabilizes HIF1α which further induces glycolysis by up-regulating the level of glucose transporters viz. GLUT-1 and glucose phosphorylating enzyme HK-II to ensure the increased flux and high retention of glucose in the cytoplasm.

**Figure 2:**
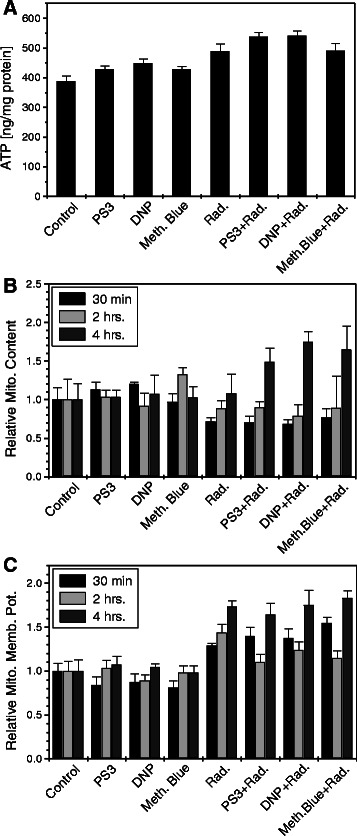
MRMs did not induce differential changes in energy and mitochondrial status in either un-irradiated or irradiated (5 Gy γ-rays) BMG-1 cells. **(A)** Shows MRMs induced glycolysis compensate the ATP production, equally in all the modifiers. **(B)** & **(C)** shows that the effects of MRMs on mitochondrial mass and membrane potential are also similar. The concentrations of different MRMs used were as follows, PS3, 25 μg/ml; DNP, 1 μM; MB, 25 μM.

MRMs inhibit the process of electron transfer and ATP generation from electron transport chain leading to incomplete respiration and reduced ATP generation. Therefore, we measured changes in ATP levels induced by MRMs (Figure 2A), besides examining the mitochondrial status by analyzing the membrane potential and mass (Figure 2B & C). Results (Figure 2) clearly show that none of the three respiratory inhibitors caused any appreciable changes in ATP (Figure 2A), while increase in mitochondrial content and related mitochondrial membrane potential was observed in cells co-treated with MRMs and radiation (only at 4 hrs. time point; Figure 2B & C). However, these changes were not significant as compared to radiation alone. These findings suggest that the significant compensatory increase in glycolysis is sufficient to meet the energy requirements in these cells. It also suggests that the effect of all the MRMs on mitochondria is similar for compensatory increase in glycolysis.

### Transient elevation of glycolysis confers radio-resistance in cells

**Figure 3:**
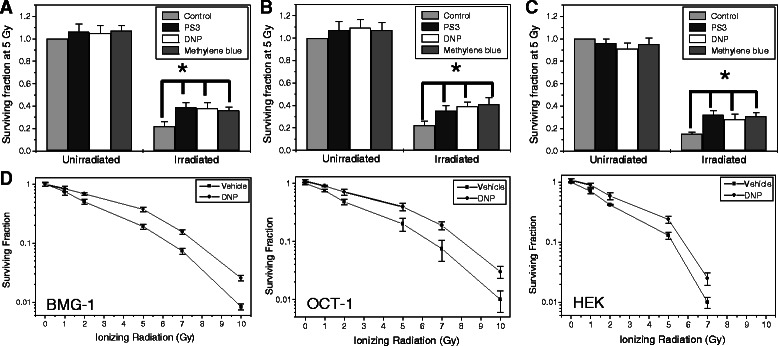
MRMs induces radio-resistance. Enhanced radio-resistance (clonogenic survival; macro-colony assay) observed due to MRMs (PS3, DNP and MB) induced glycolysis in BMG-1 **(A)**, OCT-1 **(B)** and HEK293 **(C)**. The figure **(D)** represents the dose response curve of BMG-1, OCT-1 and HEK293 cells against radiation in DNP and vehicle treated cells. The concentrations of MRMs used were as follows, PS3, 25 μg/ml; DNP, 1 μM; MB, 25 μM. After treatment cells were incubated for 4 hours in liquid holding before plating for macro colony formation. Surviving fraction of un-irradiated and irradiated samples was calculated by considering the plating efficiency of un-irradiated control as 1. The data shown are the mean values (±1 SD) of nine observations from three independent experiments. Statistical significance *p < 0.05.

To examine if transient induction of glycolysis leads to radio-resistance, we irradiated (5 Gy; γ-Rays) the cells in the presence of MRMs (that induce glycolysis) and then analyzed the clonogenicity using macro-colony assay. Indeed an increase in survival was evident in all the three cell lines, which on an average was 1.71, 1.74 and 1.9 fold in BMG-1, OCT-1 and HEK293 respectively with all the three MRMs as compared to radiation alone (Figure 3A, B and C). We further examined the effect of DNP mediated enhanced glycolysis on the radiation dose response curve in the cells and found that glycolysis stimulated cells (DNP treated) showed radio-resistance as reflected by an increase in D_1_ (dose of radiation to bring down the survival fraction to 0.37) in all the three cell lines (Figure 3D).

After establishing the “proof of concept” that elevation of glycolysis for few hours post-irradiation leads to resistance against radiation-induced cell death using more than one inducers viz. DNP, PS3 and methylene blue, we decided to investigate the mechanisms underlying this resistance using a pharmacologically more suitable molecule like DNP, as PS3 and methylene blue are associated with photo-toxicity [8,20].

### Inhibition of glycolysis reverses MRMs induced radio-resistance

**Figure 4:**
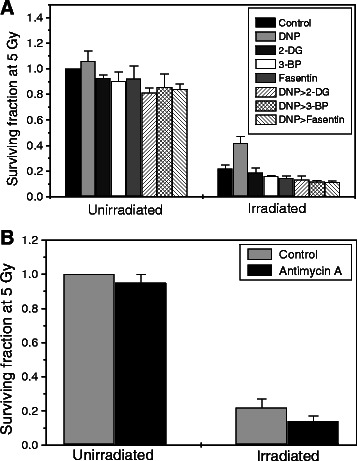
Glycolytic inhibitors reverse MRMs induced radio-resistance. Inhibition of glycolysis by 2-DG (5 mM), 3-BP (5 μM) and fasentin (25 μM) followed by DNP treatment before irradiation (5 Gy) reverses glycolysis induced radio-resistance in BMG-1 cells **(A)**. Both DNP and inhibitors of glycolysis/ glucose transporter were added simultaneously before irradiation. Inhibition of mitochondrial respiration without up-regulating glycolysis (using 5 μg/ ml Antimycin A) does not confer radio-resistance in BMG-1 cells **(B)**. The data shown are the mean values (±1 SD) of nine observations from three independent experiments. Differences were statistically significant (*p < 0.05).

To confirm that MRMs induced radio-resistance is mainly by induction of glycolysis, we inhibited the glycolysis using non-toxic concentrations of 2-deoxy-D-glucose (2-DG, hexokinase inhibitor), 3-bromo pyruvate (3-BP, hexokinase inhibitor) and fasentin (Glut-1 inhibitor) in DNP treated BMG-1 cells before exposing to radiation. All these known inhibitors of glycolysis failed to enhance the clonogenicity in DNP treated and irradiated cells as compared to radiation alone (Figure 4A). To further substantiate that MRMs induced radio-resistance is facilitated by induced glycolysis and not due to metabolic signaling associated with inhibition of mitochondrial respiration; we treated BMG-1 cells with non-toxic concentration of antimycin A (5 μg/ml), which inhibits oxidative phosphorylation but does not induce glycolysis [21]. Cells treated with antimycin A before radiation exposure did not show any increase in clonogenic survival (Figure 4B); lending further support to the notion that MRMs induced radio-resistance observed in cells is mediated by transient stimulation of glycolysis.

### Induced glycolysis facilitates DNA repair process

**Figure 5:**
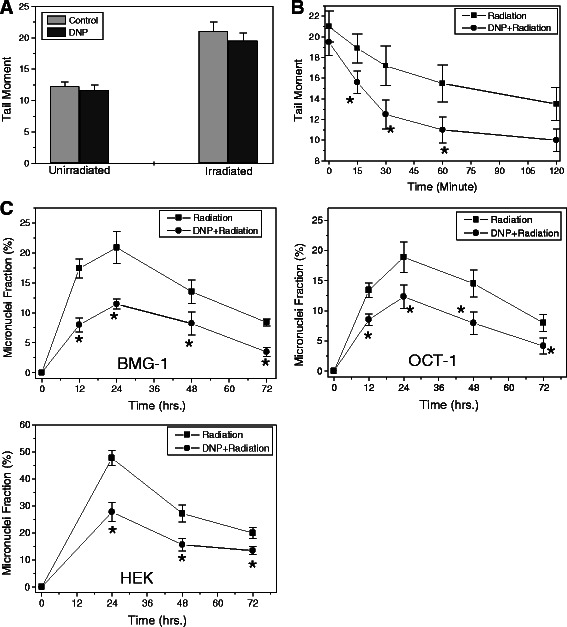
DNP induced glycolysis facilitates repair of radiation-induced DNA double strand breaks. Effects of DNP (1 μM) induced glycolysis on the induction **(A)** and kinetics of repair **(B)** of radiation (5 Gy) induced DNA damage assayed using single-cell gel electrophoresis in BMG-1 cells. **(C)** Reduction in the radiation induced cytogenetic damage (micronuclei expression) observed in glycolysis stimulated (DNP treated) BMG-1, OCT-1 and HEK293 cells. The data shown are the mean values (±1 SD) of three independent experiments. Differences were statistically significant (*p < 0.05).

Since DNA damage is one of the major contributing factor for the loss of clonogenic survival at moderate levels of absorbed radiation, we studied the effects of induced glycolysis on the induction and repair of DNA damage using single cell electrophoresis (Comet assay). We used changes in tail moment (increase and decrease) as a parameter to measure the damage induction and repair. Figure 5A shows the tail moment in BMG-1 cells, un-irradiated and immediately after irradiation. We analyzed the data using derived equations to determine the percentage of damage removed and presented in Table 1. Cells with stimulated glycolysis showed a faster kinetics of repair and the extent of damage removal at the end of 30 minutes following irradiation. The extent of damage removed was approximately 2.06 (93% / 45%) fold higher as compared to the un-stimulated cells (Figure 5B). Analysis of the repair kinetics showed that almost all damage repaired under conditions of stimulated glycolysis was handled by the fast component of repair (~93%) as compared to the un-stimulated cells (~45%). These results suggest that a higher rate as well as the extent of damage removal under conditions of enhanced glycolysis is one of the important contributing factors for enhanced resistance.

**Table 1:** **Table represents the DNA damage and repair parameters like damage induction and damage removal after 30 minutes between treated and untreated irradiated groups**

Parameters	Formula/derivation	Result
**Damage Induction, DI**	**Tail moment, TM at 0 min. – TM in un-irradiated sample**	
DI without DNP, DIc	21.2 – 12.3 (a.u.)	8.9
	(values obtained from Figure 5A)	
DI for DNP treated, DId	19.6 – 12.0 (a.u.)	7.6
	(values obtained from Figure 5A)	
**% Damage removed at 30 min., DR**_**30min.**_	**[(TM**_**0min.**_ **– TM**_**30min**_ **)/DI] x 100**	
DR without DNP, DRc_30min_	[(21.2 – 17.2)/8.9] x 100 = [0.45] x 100	45%
	(values obtained from Figure 5A & B)	
DI for DNP treated, DRd_30min_	[(19.6 – 12.5)/7.6] x 100 = [0.93] x 100	93%
	(values obtained from Figure 5A & B)	
**Fold change in damage removed at 30 min.**	**DRd**_**30min/**_ **DRc**_**30min**_	
	93/45	2.06

The values were obtained from Figure 5 A & B and applied in the derived formulas to obtain the final fold change of damage removed.

Because, radiation predominantly causes mitotic catastrophe (an event in which a cell is destroyed during mitosis) linked death at moderate doses [22], we investigated radiation induced cytogenetic damage, which is involved in mitotic catastrophe. Cytogenetic damage was assessed by counting cells with micronucleus, which arises from unrepaired/mis-repaired DNA double strand breaks (DSBs) following irradiation and correlates with changes in survival [23]. The kinetics of micronuclei expression followed until 72 h post-irradiation clearly showed a significant decrease in the fraction of cells with micronuclei in glycolysis stimulated BMG-1, OCT-1 and HEK293 cells at all time points (Figure 5C) suggesting reduced residual DNA damage, in line with the enhanced repair observed (Figure 5B).

### Induced glycolysis facilitates NHEJ and HR pathways of DNA repair

**Figure 6:**
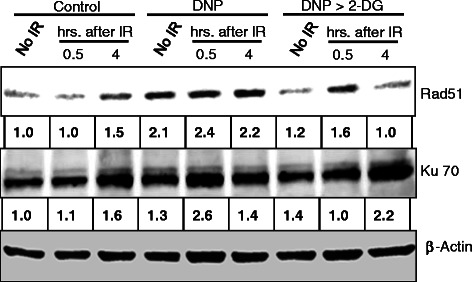
Time dependent changes in the levels of DNA repair proteins (Rad51 and Ku-70) observed following irradiation (5 Gy) showing the effects of DNP (1 μM) and DNP > 2-DG (5 mM) in BMG-1 cells. Numbers shown below the bands in the western blots represent the values normalized with respective β-actin using densitometry on Imagequant 5.2 program and represents the fold change relative to unirradiated control of each group.

DSBs are particularly deleterious to cells and its inefficient repair can lead to cell death. DSBs in the mammalian genome are repaired through homologous recombination (HR) and non-homologous end joining (NHEJ) repair pathways. Rad51 is a critical component of the HR pathway whereas; Ku-70 and Ku-80 are critical component of NHEJ pathway [24]. To examine if a particular DSB repair pathway is facilitated under the conditions of induced glycolysis, we examined the status of DSB repair initiating proteins, Rad51 and Ku-70 involved in HR and NHEJ repair pathways, respectively. Western blot analysis carried out in BMG-1 cells showed that the level of Rad51 protein increased approximately 1.5 fold at 4h following irradiation (5 Gy; Figure 6). However, in DNP treated cells, an early 2 fold increase was noted in the level of Rad51 at 0.5 h after irradiation (Figure 6), suggesting a rapid induction of repair and faster removal of DSB leading to lesser residual DNA damage in glycolysis stimulated cells. Similar results were obtained for time dependent changes in Ku-70 levels, which was also found to be up-regulated by 2 folds, 0.5 hr after irradiation, in DNP treated cells (Figure 6A). Although, increase in Rad51 level correlates with the faster repair kinetics observed here, increase in the level of Rad51 following irradiation are at variance with earlier observations [25,26]. These observations are in line with our observations of a faster rate of DNA break rejoining (Figure 5B), wherein the damage (tail moment) returned close to the basal level within 0.5 h following irradiation in glycolysis stimulated cells (Figure 5B). Inhibition of glycolysis using 2-DG in DNP stimulated cells did not show appreciable increase in the level of these repair proteins Rad51 and Ku70, lending support to the proposition that stimulation of glycolysis enhances the kinetics of DNA repair by increasing the expression of repair proteins Rad51 and Ku70. These results suggest that both HR and NHEJ pathways of DNA repair are operationally efficient following stimulation of glycolysis, thereby leading to a faster damage removal and enhanced survival. Taken together, these observations suggest that DNP induced transient elevation of glycolysis results in activation of both HR and NHEJ pathway to facilitate the DNA repair thereby making the cells relatively more resistant.

## Discussion

Irradiation of cells causes macromolecular damage (viz DNA, protein etc) stimulating multiple signaling pathways viz DNA repair, cell cycle check points, apoptosis and senescence that collectively determine the fate of cells [22]. It is well established that at moderate absorbed doses of low LET radiation (like gamma rays and X-rays), the main contributing factor responsible for loss of clonogenecity (cell survival) is the mitotic death linked to cytogenetic damage that arises from residual DNA damage [22,27]. Incomplete repair and/or mis-repair of DNA strand breaks results in chromosomal damage that can be observed as various chromosomal aberrations in the metaphase of the irradiated population and as micronuclei in the daughter cells [27], although mitotic spindle dysfunction and other disturbances also lead to micronuclei formation and related nuclear damage [28]. Therefore, alterations in the DNA repair processes that are operative for few hours following irradiation are expected to influence the cell survival as well as the level of cytogenetic damage. In the present study, we observed an increase in the clonogenic survival following transient stimulation of glycolysis (Figure 3) that correlated with the decrease in the level of micronuclei expression (Figure 5C) suggesting a reduction in the residual DNA damage under these conditions. While stimulation of glycolysis did not significantly alter the level of induction of DNA damage (Figure 5A), the rate of DNA strand break rejoining as well as the extent of damage removal were clearly higher that resulted in a decrease in the residual DNA damage at the end of 30 minutes after irradiation (Figure 5B).

It will be interesting to see whether the mechanisms underlying radio-resistance seen in cancer cells, where a stable phenotype with enhanced glycolysis gradually develops during tumorigenesis will be identical to the transient stimulation of glycolysis observed here. In this respect, it is pertinent to note that inhibition of mitochondrial respiration [29] that leads to enhanced glycolysis (as seen here; Figure 1) often results in the stimulation of HIF1α expression of many genes in the glycolytic pathway and up-regulated in malignantly transformed cells and tumors [30,31]. Stimulation of glycolysis seen as a compensatory mechanism following a fall in the mitochondrial ATP production induced by respiratory inhibitors has been suggested to be due to the activation of AMP kinase triggered by an increase in the AMP level (and AMP/ATP ratio) due to ATP breakdown [29]. Some recent evidences suggest that inhibition of mitochondrial respiration leads to accumulation of glycolytic end products like pyruvate and lactate which could dramatically increase HIF1α accumulation in cancer cells by inhibiting the prolyl hydroxylase enzyme activity. Moreover, other mediators like the expression of TKTL1 (Transketolase-like 1, an enzyme of pentose phosphate pathway) in head and neck carcinomas and gliomas leads to metabolic switch by stabilizing HIF1α for improving energy yield from glucose via glycolysis and enhancing antioxidant defence against ROS via pentose phosphate pathway [32,33]. In this respect it is pertinent to note that the increase in the protein levels of regulators of glycolysis stimulated by DNP in BMG-1 cells viz. Glut-1 and HK-II observed here (Figure 1D) are all regulated by HIF1α [9,27]. Therefore, radio-resistance following DNP induced transient elevation in glycolysis appears to partly involve similar mechanisms that are reported as activators of glycolysis in cancer cells generally associated with resistance to radiation and other drugs. Further, sensitization of BMG-1 cells to radiation in the presence of Antimycin A (Figure 4B), which inhibits mitochondrial respiration without compensatory increase in glycolysis and the glycolytic inhibitor 2-DG strongly suggests that MRMs (DNP, PS3 and MB) induced radio-resistance in cells is mainly due to increase in glycolysis and not because of inhibition of mitochondrial respiration.

DNA double strand breaks, the most lethal lesions widely considered to be responsible for radiation-induced cell death are repaired by both homologous recombination (HR) and non-homologous end joining (NHEJ) in a proliferating mammalian cell population. Rad51 is a critical component of DNA DSB repair pathway [34], which is widely reported to redistribute within the nucleus following DNA damage suggesting the formation of repair foci involving this recombinase [25,26,35], although radiation induced elevated level of this protein enhances radio-resistance has also been reported [35]. Rapid increase in the level of Rad51 protein at 30 minutes following irradiation in glycolysis stimulated (DNP treated) cells observed here (Figure 6) is similar to the observations in the radio-resistant spheroids of DU145 carcinoma cell line [36]; which also interestingly has a significant level of HIF1α with elevated glycolysis and increased resistance to radiation [37]. Increased level of Rad51 following irradiation seen here, particularly in DNP treated cells is at variance with earlier reports [25,26,35] and may arise due to many reasons viz. proteasomal degradation, altered metabolic status of the cells [36,38] or changes in the interactions with other members of the repair complex thereby altering the immune reactivity, which needs further investigations. A profound increase in the level of Ku-70 (Figure 6) in DNP treated cells also facilitates a faster repair by NHEJ conferring resistance against radiation in high glycolytic cells. Increased level of Ku-70 has been reported to increase cellular tolerance against ischemic stress [39] and adaptation to ischemia provides hypoxia mediated *in-vivo* tumor radio-resistance [40]. Our results also suggest that increased Ku-70 level may facilitate NHEJ pathway of DSB repair in high glycolytic cells leading to reduced micronuclei and increased cell survival against radiation. Some recent evidences suggest that DNA damage induced by adriamycin enhances the TIGAR and TKTL1 expression and knocking down the TKTL1 or WRN complex both leads to reduced glycolytic metabolism and accumulation of DNA damage in cancer cells [33,41]. Although these observations strongly suggest enhanced repair of radiation-induced DNA DSBs by stimulated glycolysis, underlying mechanisms responsible for this can only be unraveled using studies with DNA repair deficient and efficient cell systems following the stimulation of glycolysis. Pending this insight to be unraveled, results of the present studies lend support to our hypothesis that enhanced glycolysis is a favorable metabolic change that facilitates DNA repair, which appears to be partly responsible for radio-resistance, which is often observed in cancer cells.

The transient increase in glycolysis conferring resistance against radiation-induced cell death in normal HEK293 cells (clonogenic survival; Figure 3C) has important implications in radiation countermeasure, as pharmacological agents that stimulate glycolysis at the systemic level may act as radio-protective agents. Indeed, administration of low amounts of, DNP used in this study for stimulating glycolysis has been shown to be safe for humans/canine [42]. Facilitated DNA repair leading to reduced cytogenetic damage and mitotic linked cell death along with upregulated level of HK-II and Ku-70 which are generally elevated with the increase in glycolysis is known to inhibit intrinsic pathway of apoptosis [43] and therefore can protect both the hematopoietic and Gastro Intestinal system.

## Conclusion

In the present study, we show that transient induction of glycolysis by respiratory inhibitors gives rise to radio-resistance by activating both the NHEJ and HR DNA repair pathways, thereby reducing residual DNA damage and cytogenetic damage linked mitotic death. Further understanding of the mechanisms underlying glycolysis induced facilitated DNA repair and radio-resistance may help in unraveling critical molecular targets responsible for resistance and facilitate the design and/or identification of molecules/agents that specifically overcome resistance linked to enhanced glycolysis, thereby enhancing the efficacy of radio- and chemotherapies.
